# A prediction model for lymph node metastases using pathologic features in patients intraoperatively diagnosed as stage I non-small cell lung cancer

**DOI:** 10.1186/s12885-017-3273-x

**Published:** 2017-04-13

**Authors:** Fei Zhao, Yue Zhou, Peng-Fei Ge, Chen-Jun Huang, Yue Yu, Jun Li, Yun-Gang Sun, Yang-Chun Meng, Jian-Xia Xu, Ting Jiang, Zhi-Xuan Zhang, Jin-Peng Sun, Wei Wang

**Affiliations:** Department of Thoracic Surgery, First Affiliated Hospital of Nanjing Medical University, 300 Guangzhou Road, Nanjing, 210029 China

**Keywords:** Non-small-cell lung cancer, Lymph node, Metastasis, Multivariable logistic model

## Abstract

**Background:**

There is little information on which pattern should be chosen to perform lymph node dissection for stage I non-small-cell lung cancer. This study aimed to develop a model for predicting lymph node metastasis using pathologic features of patients intraoperatively diagnosed as stage I non-small-cell lung cancer.

**Methods:**

We collected pathology data from 284 patients intraoperatively diagnosed as stage I non-small-cell lung cancer who underwent lobectomy with complete lymph node dissection from 2013 through 2014, assessing various factors for an association with metastasis to lymph nodes (age, gender, pathology, tumour location, tumour differentiation, tumour size, pleural invasion, bronchus invasion, multicentric invasion and angiolymphatic invasion). After analysing these variables, we developed a multivariable logistic model to estimate risk of metastasis to lymph nodes.

**Results:**

Univariate logistic regression identified tumour size >2.65 cm (*p* < 0.001), tumour differentiation (*p* < 0.001), pleural invasion (*p* = 0.034) and bronchus invasion (*p* < 0.001) to be risk factors significantly associated with the presence of metastatic lymph nodes. On multivariable analysis, only tumour size >2.65 cm (*p* < 0.001), tumour differentiation (*p* = 0.006) and bronchus invasion (*p* = 0.017) were independent predictors for lymph node metastasis. We developed a model based on these three pathologic factors that determined that the risk of metastasis ranged from 3% to 44% for patients intraoperatively diagnosed as stage I non-small-cell lung cancer. By applying the model, we found that the values ŷ > 0.80, 0.43 < ŷ ≤ 0.80, ŷ ≤ 0.43 plus tumour size >2 cm and ŷ ≤0.43 plus tumour size ≤2 cm yielded positive lymph node metastasis predictive values of 44%, 18%, 14% and 0%, respectively.

**Conclusions:**

A non-invasive prediction model including tumour size, tumour differentiation and bronchus invasion may be useful to give thoracic surgeons recommendations on lymph node dissection for patients intraoperatively diagnosed as Stage I non-small cell lung cancer.

**Electronic supplementary material:**

The online version of this article (doi:10.1186/s12885-017-3273-x) contains supplementary material, which is available to authorized users.

## Background

Lung cancer is the leading cause of cancer death worldwide [1] and metastasis to lymph nodes directly determines the stage and prognosis of this disease. Computed tomography (CT) remains the most widely used tool for assessment of the tumour and lymph node involvement in patients with early-stage non-small-cell lung cancer (NSCLC) [2–5]. In general, lymph nodes with short-axis diameters of >1 cm seen on CT scan are considered metastatic. Unfortunately, the accuracy of CT scan for preoperative lymph node stage is only 45%–79% [2–6]. In addition, studies have demonstrated that 12%–17% of patients histologically confirmed as N2 are preoperatively diagnosed as N0 because their CT scan results showed the involved lymph nodes to have short-axis diameters of <1 cm [4, 5, 7]. Many other methods of preoperative N-staging, e.g. positron emission tomography, mediastinoscopy and endoscopic ultrasound-guided fine-needle aspiration, are not routinely used for patients with clinical stage I disease. In addition, these methods yield a considerable number of false-negative results [8–10].

There is ample high-quality evidence on the advantages of lymph node dissection in lung cancer surgery, including the American College of Surgeons Oncology Group (ACOSOG) Z0030 trial [11], although the benefits of complete lymph node dissection for patients with stage I NSCLC are still controversial [12–14]. There is little information on which pattern should be chosen to perform lymph node dissection for patients intraoperatively diagnosed as stage I non-small-cell lung cancer. A non-invasive prediction model that is able to predict lymph node metastasis would allow surgeons to make appropriate decisions on the extent of the dissection, removing lymph nodes that are most likely to contain metastases, while avoiding unnecessary tissue damage in order to accelerate patients’ postoperative recovery.

The goal of this study was to identify risk factors that would predict differences in lymph node metastasis and to develop a scoring system to predict the presence of lymph node metastasis. The aim is to determine the appropriate pattern of lymph node dissection for various patients intraoperatively diagnosed as stage I NSCLC.

## Methods

### Patient selection

A total of 284 consecutive patients who underwent surgical resection for primary lung cancer at our hospital from January 2013 to December 2014 were reviewed retrospectively. The records of patients intraoperatively diagnosed as stage I NSCLC who underwent lobectomy with complete lymph node dissection according to the lymph node nomenclature were selected for this study. All patients met the criteria for stage I NSCLC based on the new International Staging System for NSCLC (National Comprehensive Cancer Network (NCCN) Guidelines Version 3.2014: Staging Non-Small Cell Lung Cancer) [15]. We excluded patients from this study who met any one of the following conditions: 1) tumour size > 4 cm and lymph node > 1 cm at the largest diameter on CT imaging or evidence of distant metastasis; 2) preoperative chemotherapy or radiotherapy; 3) previous or coexistent tuberculosis or malignant disease; 4) complete lymph node dissection that did not meet the current standards (i.e. all lymph node stations, including right-hand stations 2–4 and 7–9 and left-hand stations 2–9); 5) pure ground-glass opacity on CT imaging; 6) synchronous lung cancers, 7) sublobar resection, segmentectomy or partial resection or 8) Intraoperative frozen rapid pathological results showed tumour size > 4 cm in the largest diameter.

Patients were preoperatively assessed with chest x-ray, chest and upper abdominal CT scan, brain magnetic resonance imaging and bone scintigraphy. CT scan was used for preoperative N-staging. The surgical approach for primary lung cancer resection was via video-assisted thoracic surgery.

### Statistical analysis

The baseline patient characteristics were summarized in percentages for categorical variables and as mean ± SD (Standard Deviation) for continuous variables. The chi-square test and Fisher’s exact tests were used to analyse differences in these percentages between the groups. Differences between the groups were analysed using the Kruskal–Wallis test. Significance of associations with the outcome of nodal metastases was first evaluated using a univariate logistic analysis. Those significant variables were analysed by multivariable analysis as independent predictors for lymph node metastasis. Odds ratios (ORs) with 95% confidence intervals (CIs) were calculated. Clinically relevant variables obtained by multivariable analysis were included in the multivariable model. The resulting model coefficients were applied to the cohort to calculate predicted values from the logistic equation: ŷ = 1/[1 + exp. (−xβ)]. All confidence intervals, significance tests and resulting *P* values were two-sided, with an alpha level of 0.05. Statistical analyses were performed using STATA software, release 13.

## Results

### Patient characteristics and prevalence of lymph node metastasis

A total of 284 patients intraoperatively diagnosed as stage I NSCLC were included in this study. Table 1 shows the patients’ demographics and clinical characteristics. The mean age was 60.78 years (range 31–83). Histologically, the tumours in 248 patients (87%) were identified as adenocarcinoma and in 36 (13%) as squamous cell carcinoma. The tumour originated in the right upper lobe in 82 patients (29%), right middle lobe in 16 (6%), right lower lobe in 39 (14%), left upper lobe in 77 (27%), left lower lobe in 51 (18%) and in mixed lobes in 19 (6%). Mean tumour size was 2.44 cm (range from 0.4 to 4 cm). The tumour differentiation included I (86 patients, 30%), II (176 patients, 62%), III (22 patients, 8%). Pleural invasion was present in 64 patients (23%) and bronchus invasion in 37 (13%).

**Table 1:** Patient Demographics and Clinical Characteristics

Variables	Value
Number	284
Age (years)	
Mean ± SD (range)	60.78 ± 9.2 (31–83)
Gender (%)	
Male	144 (51%)
Female	140 (49%)
Pathology	
Squamous cell carcinoma	36 (13%)
Adenocarcinoma	248 (87%)
Tumor location (%)	
Right Upper Lobe	82 (29%)
Right Middle Lobe	16 (6%)
Right Lower Lobe	39 (14%)
Left Upper Lobe	77 (27%)
Left Lower Lobe	51 (18%)
Mixed lobes	19 (6%)
Differentiation (%)	
I	86 (30%)
II	176 (62%)
III	22 (8%)
Tumor size (cm)	
Mean ± SD (range)	2.44 ± 0.97 (0.4-4 cm)
Pleura invasion	
Absent	220 (77%)
Present	64 (23%)
Bronchus invasion	
Absent	247 (87%)
Present	37 (13%)
Multicentric invasion (%)	
Absent	264 (93%)
Present	20 (7%)
Angiolymphatic invasion (%)	
Absent	274 (96%)
Present	10 (4%)
Neural invasion	
Absent	283 (100%)
Present	1 (0%)

*SD* standard deviation

Lymph node metastases were not found in 215 patients (group I) but were present in 69 (group II) (Table 2). The characteristics in these two groups were compared in terms of age, gender, pathology, tumour location, tumour differentiation, tumour size, pleural invasion, bronchus invasion, multicentric invasion, neural invasion and angiolymphatic invasion. Compared with group I, group II had a significantly larger tumour size than that in group I (2.92 ± 0.87 vs. 2.28 ± 0.95, *P* < 0.001). There were significant statistical differences between the groups by the χ^2^ test in terms of tumour differentiation (I, II, III) (*P* < 0.001), bronchus invasion (absent vs. present) (*P* < 0.001) and pleural invasion (absent vs. present) (*P* = 0.033).

**Table 2:** Demographics of patients in the Negative lymph Node Metastases (LNM) and Positive LNM groups

Variables	Group		*P* value
	Negative LNM	Positive LNM	
Number	215	69	
Age (years)			0.118
Mean ± SD	61.27 ± 9.38	59.28 ± 8.49	
Gender			0.997
Male	109	35	
Female	106	34	
Pathology			0.176
Squamous cell carcinoma	24	12	
Adenocarcinoma	191	57	
Tumor location			0.368
Right Upper Lobe	62	20	
Right Middle Lobe	14	2	
Right Lower Lobe	28	11	
Left Upper Lobe	63	14	
Left Lower Lobe	34	17	
Mixed lobes	14	5	
Differentiation			<0.001^*^
I	80	6	
II	119	57	
III	16	6	
Tumor size (cm)			<0.001
Mean ± SD	2.28 ± 0.95	2.92 ± 0.87	
Pleura invasion			0.033^*^
Absent	173	47	
Present	42	22	
Bronchus invasion			<0.001^*^
Absent	196	51	
Present	19	18	
Multicentric invasion			1 (Fish)
Absent	200	64	
Present	15	5	
Angiolymphatic invasion			0.263 (Fish)
Absent	209	65	
Present	6	4	
Neural invasion			1.0 (Fish)
Absent	214	69	
Present	1	0	

*SD* standard deviation

*P < 0.05

**Fig. 1:**
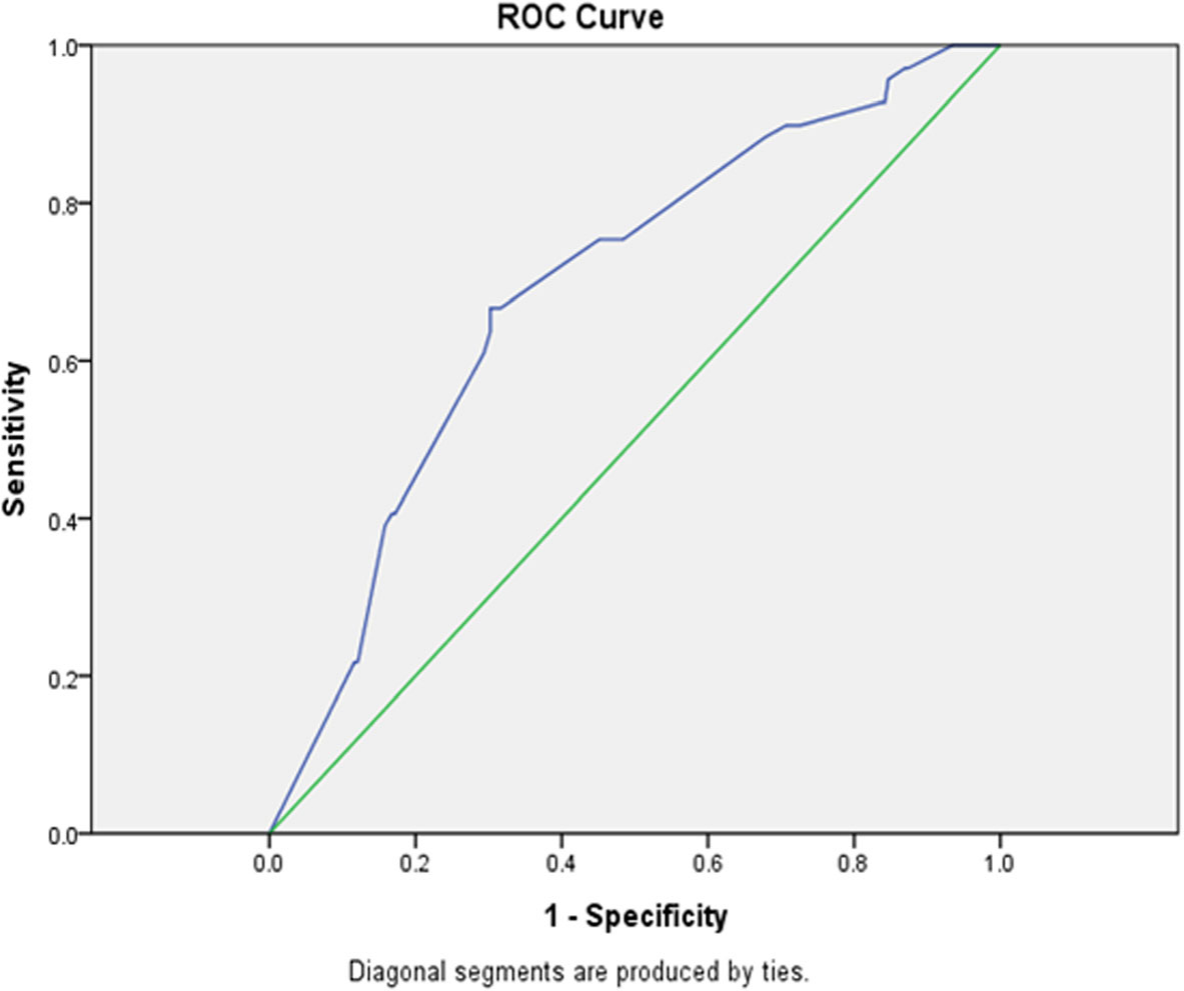
The ROC (Receiver Operating Characteristic) curve of tumor size between group I and group II

To evaluate the predictive value of tumour size between the groups, we used Receiver Operating Characteristic (ROC) curve analysis. As shown in Fig. 1, the area under the ROC curve for tumour size between group I and group II was 0.691 (95% CI: 0.621–0.761; *P* < 0.001); the optimal cut-off value was 2.650 cm (sensitivity: 67%; specificity: 70%; Youden’s index: 0.364).

### Association of Individual Pathologic Characteristics with Nodal Metastasis

Univariate analysis showed that tumour size greater than 2.650 cm (OR =4.62, 95% CI 2.59–8.24; *P* < 0.001), tumour differentiation (I vs II + III, OR =6.22, 95% CI 2.58–15.03; *P* < 0.001), pleural invasion (absent vs present, OR =1.93, 95% CI 1.05–3.54; *P* = 0.034) and bronchus invasion (absent vs present, OR =3.64, 95% CI 1.78–7.44; *P* < 0.001) were the four significant risk factors associated with the presence of metastatic lymph nodes (Table 3).

**Table 3:** Univariate analysis of the risk factors for lymph node metastases

Variables	OR (95% CI)	*P* value
Age		
≤ 60 vs >60	0.75 (0.44–1.30)	0.304
Gender		
male vs female	1.0 (0.58–1.72)	0.997
Pathology		
Squamous cell carcinoma VS Adenocarcinoma	0.60 (0.28–1.27)	0.179
Tumor location		
Right lobes vs Left lobes	1.00 (0.57–1.77)	0.98
Upper lobes vs Middle +Left lobes	1.45 (0.82–2.56)	0.199
Single lobes vs Mixed lobes	1.12 (0.39–3.24)	0.832
Differentiation		
I VS II + III	6.22 (2.58–15.03)	<0.001^*^
Tumor size		
≤ 2.65 cm vs >2.65 cm	4.62 (2.59–8.24)	<0.001^*^
Pleura invasion		
Absent vs Present	1.93 (1.05–3.54)	0.034^*^
Bronchus invasion		
Absent vs Present	3.64 (1.78–7.44)	<0.001^*^
Multicentric invasion		
Absent vs Present	1.04 (0.36–2.98)	0.939
Angiolymphatic invasion		
Absent vs Present	2.14 (0.59–7.83)	0.249

^*^ *P* < 0.05

### Multivariable analysis of pathologic characteristics associated with nodal metastasis

Multivariate analysis of the four risk factors obtained on univariate analysis showed that only the tumour size (≤2.65 cm vs. >2.65 cm, OR =3.23, 95% CI 1.75–5.93; *P* < 0.001), tumour differentiation (I vs II + III, OR =3.64, 95% CI 1.44–9.16; *P* = 0.006) and bronchus invasion (absent vs. present, OR =2.54, 95% CI 1.18–5.46; *P* = 0.017) were independent predictors associated with the presence of metastatic lymph nodes. However, pleural invasion (absent vs. present, OR =1.64, 95% CI 0.84–3.21; *P* = 0.146) was not a significant predictor of lymph node metastasis (Table 4).

**Table 4:** Multivariate analysis of the risk factors for lymph node metastases

Variables	β	OR (95% CI)	*P* value
Differentiation			
I VS II + III	1.291	3.64 (1.44–9.16)	0.006^*^
Tumor size			
≤ 2.65 cm vs >2.65 cm	1.171	3.23 (1.75–5.93)	<0.001^*^
Pleura invasion			
Absent vs Present	0.496	1.64 (0.84–3.21)	0.146
Bronchus invasion			
Absent vs Present	0.931	2.54 (1.18–5.46)	0.017^*^
Intercept	−3.013		

^*^ *P* < 0.05

### Multivariable logistic regression model derivation and development

On multivariable analysis, only three covariates remained in the final model. Using these three variables (Table 5), a scoring system was developed to discriminate between patients with and without lymph node metastasis. The risk scores for individual patients were calculated using the following formula: xβ = −2.947 + (1.368 × Differentiation (I vs. II + III, *I* = 0, II + III = 1)) + (1.188 × Tumour Size (2.65 cm vs. >2.65 cm, ≤2.65 cm = 0, >2.65 cm = 1)) + (0.876 × Bronchus Invasion (absent =0, present =1)).

**Table 5:** Multivariate analysis of the risk factors for development of model

Variables	β	OR (95% CI)	*P* value
Differentiation			
I VS II + III	1.368	3.93 (1.57–9.83)	0.003^*^
Tumor size			
≤ 2.65 cm vs >2.65 cm	1.188	3.28 (1.79–6.01)	<0.001^*^
Bronchus invasion			
Absent vs Present	0.876	2.40 (1.13–5.13)	0.023^*^
Intercept	−2.947		

^*^ *P* < 0.05

The probabilities of lymph node metastasis were calculated using the following formula (ŷ = 1/[1 + exp.(−xβ)]): ŷ = 1/[1 + exp. (2.947 - (1.368 × Differentiation (I vs. II + III, *I* = 0, II + III = 1)) - (1.188 × Tumour Size (≤2.65 cm vs. >2.65 cm, ≤2.65 cm = 0, >2.65 cm = 1)) - (0.876 × Bronchus Invasion (absent =0, present =1))].

### Model performance and selecting cut-off values to discriminate patients with lymph node metastasis

**Fig. 2:**
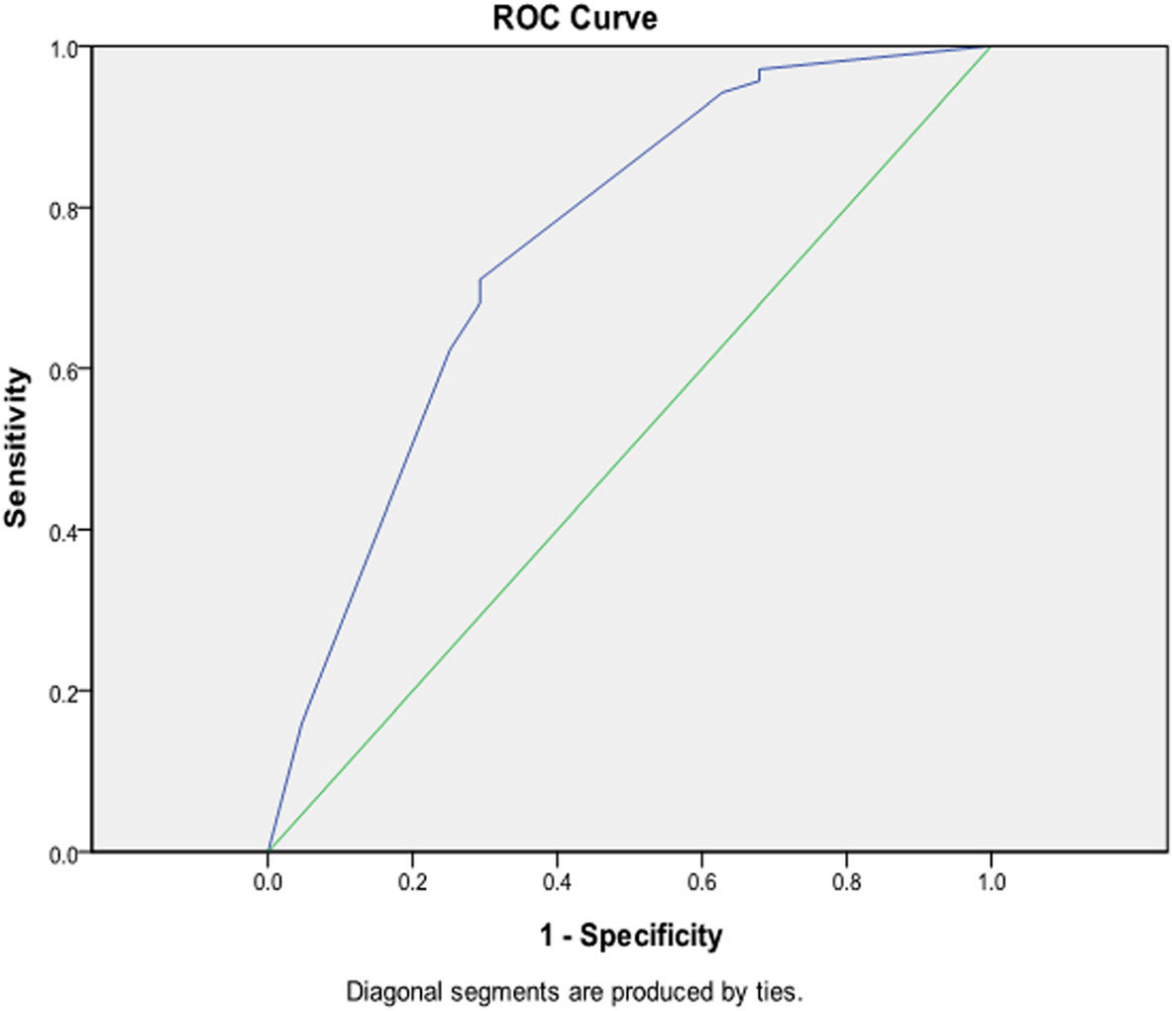
The ROC (Receiver Operating Characteristic) curve of the selected model

As shown in Fig. 2, the area under the ROC curve of the selected model was 0.753 (95% CI 0.692–0.814, standard error 0.031) and the optimal cut-off value was 0.7997 ≈ 0.80 (sensitivity: 71%, specificity: 71%, Youden’s index: 0.417). In all patients, using a score threshold of ≤0.80, 20 (12%) of 172 patients with lymph node metastasis were correctly identified, whereas 152 (88%) of 172 without lymph node metastasis were correctly identified. Using a score threshold of >0.80, 49 (44%) of 112 patients with lymph node metastasis were correctly identified, whereas 63 (56%) of 112 without lymph node metastasis were correctly identified.

When all three covariates (tumour size, tumour differentiation, bronchus invasion) were equal to zero, we found that the cut-off value was 0.42685 ≈ 0.43. In all patients, using a score threshold of ≤0.43, 2 (3%) of 71 patients with lymph node metastasis were correctly identified, whereas 69 (97%) of 71 without lymph node metastasis were correctly identified. Using a score threshold of >0.43, 67 (31%) of 213 patients with lymph node metastasis were correctly identified, whereas 146 (69%) of 213 without lymph node metastasis were correctly identified.

Using a score threshold between 0.43 and 0.80, 18 (18%) of 101 patients with lymph node metastasis were correctly identified, whereas 83 (82%) of 101 without lymph node metastasis were correctly identified. So, we obtained three score thresholds, ŷ ≤ 0.43, 0.43 < ŷ ≤ 0.80 and ŷ > 0.80.

## Discussion

A complete lymph node dissection, removing all ipsilateral lymph nodes which can be seen at operation [16], can provide more accurate pathologic staging and better clinical outcomes for some patients. It is considered a standard surgical treatment for patients diagnosed preoperatively with lymph node metastases. However, complete lymph node dissection is not regarded as a routine surgical procedure for patients intraoperatively diagnosed as stage I NSCLC, as some studies have demonstrated a lack of significant differences in outcome between selective lymph node sampling and complete lymph node dissection in patients with early-stage lung cancer [13, 17].

However each patient exhibits different clinical characteristics that affect the risk of lymph node metastasis in early-stage lung cancer. In this study, we collected pathology data from 284 patients intraoperatively diagnosed as stage I NSCLC who underwent lobectomy with complete lymph node dissection and investigated factors that might be associated with metastasis to lymph nodes (age, gender, pathology, tumour location, tumour differentiation, tumour size, pleural invasion, bronchus invasion, multicentric invasion and angiolymphatic invasion).

First, we used univariate analysis to find associations between pathologic factors and lymph node metastasis. The results showed that only the tumour size (>2.65 cm), tumour differentiation, pleural invasion and bronchus invasion were significant risk factors. The other factors tested, including age, gender, pathologic type, tumour location, multicentric invasion, angiolymphatic invasion and neural invasion were excluded as risk factors associated with lymph node metastasis.

Furthermore, multivariate analysis of the four risk factors identified on univariate analysis found that only tumour size (>2.65 cm), tumour differentiation and bronchus invasion were independent predictors of lymph node metastasis. Pleural invasion was excluded as an independent predictor in this analysis.

These three independent predictors were kept in the final model. After developing the multivariable logistic regression model, we finally obtained three score thresholds, ŷ ≤0.43, 0.43 < ŷ ≤ 0.80 and ŷ > 0.80 (Table 6). As shown in the table, we found that when ŷ was ≤0.43, patients with lymph node metastasis accounted for 3% of all patients, and when ŷ was ≤0.43 and tumour size was ≤2 cm, no patients had lymph node metastasis. However, when ŷ was ≤0.43 and tumour size was >2 cm, the percentage of patients identified with lymph node metastasis increased to 14%. With 0.43 < ŷ ≤ 0.80, patients with lymph node metastasis accounted for 18% of all patients. When ŷ was >0.80, the patients with lymph node metastasis accounted for 44% of all patients.

**Table 6:** Analysis of lymph Node Metastases (LNM)

Variables	ŷ ≤ 0.43			0.43 ~ 0.80			ŷ > 0.80		
	Negative LNM	Positive LNM (%)	Total	Negative LNM	Positive LNM (%)	Total	Negative LNM	Positive LNM (%)	Total
Num	69	2(3)	71	83	18(18)	101	63	49(44)	112
Differentiation									
I	69	2(3)	71	11	2(15)	13	0	2(100)	2
II + III	−	−	−	72	16(18)	88	63	47(43)	110
Tumor size(cm)									
≤ 2	57	0(0)	57	50	13(20)	64	4	4(50)	8
2 ~ 2.65	12	2(14)	14	22	4(15)	26	5	0(0)	5
> 2.65	−	−	−	11	1(8)	12	54	45(45)	99
Bronchus invasion									
Absent	69	2(3)	71	83	17(17)	100	44	32(42)	76
Present	−	−	−	0	1(100)	1	19	17(47)	36

Thus we demonstrated that lymph node dissection is not necessary for those patients intraoperatively diagnosed as stage I NSCLC whose ŷ value obtained from the model is less than or equal to 0.43 and whose tumour size is ≤2 cm. Complete lymph node dissection or lymph node sampling would be appropriate if the ŷ value from the model is less than or equal to 0.43 but the tumour size is >2 cm or if ŷ is more than 0.43 and less than or equal to 0.80. Complete lymph node dissection must be performed for patients whose ŷ value obtained from the model is more than 0.80.

However, our study has some limitations. This study was conducted at a single institution with retrospective methods and demonstrated the necessity of further prospective study. Further prospective study with multicenter trial should be performed to comprehensively evaluate this model for prediction of lymph node metastases in patients intraoperatively diagnosed as Stage I non-small cell lung cancer.

## Conclusions

After a comprehensive analysis of our results concerning various clinical factors, we conclude that the incidence of lymph node metastasis would be lowest when we obtained a ŷ value from the model less than or equal to 0.43 along with a tumour size ≤2 cm. For other patients intraoperatively diagnosed as stage I NSCLC, the risk of lymph node lymph node metastasis was greater, so that and complete lymph node dissection or lymph node sampling is necessary.

## Additional file

Additional file 1: Support file containing the Age ranges, Pathology, location, Differentiation, Tumor size 2.65 cm, Pleura invasion, Bronchus invasion, Multicentric invasion, Angiolymphatic invasion, Neural invasion and LNM (lymph node metastasis) described in categorical variables and Tumorsize, xβ and ŷ described in continuous variables. (XLSX 32 kb)

## Abbreviations

ACOSOG: American College of Surgeons Oncology Group
CT: Computed tomography
NSCLC: Non-small-cell lung cancer
ROC: Receiver Operating Characteristic
SD: Standard Deviation
